# The Phylogeography and Diversification of an Endemic Trapdoor Spider Genus, *Stasimopus* Simon 1892 (Araneae, Mygalomorphae, Stasimopidae) in the Karoo, South Africa

**DOI:** 10.1002/ece3.70621

**Published:** 2024-11-24

**Authors:** Shannon Brandt, Robin Lyle, Catherine Sole

**Affiliations:** ^1^ Department of Zoology and Entomology University of Pretoria Pretoria South Africa; ^2^ INRAE, UMR BIOGECO Bordeaux France; ^3^ Agricultural Research Council – Plant Health and Protection, Biosystematics Pretoria South Africa

**Keywords:** divergence dating, Karoo, Mygalomorphae, phylogeography, South Africa, *Stasimopus*

## Abstract

The genus *Stasimopus* is endemic to South Africa but has never undergone a phylogeographic review. This study aims to unravel the phylogeographic patterns and history of the many *Stasimopus* species which occur in the greater Karoo region. A fossil‐calibrated phylogeny was produced based on three gene regions (CO1, 16S and EF‐1ɣ) for *Stasimopus* (Cor‐k‐lid trapdoor spiders) specimens collected in the Karoo region, to infer dates of origin and diversification. Demographic analyses were performed on species with sufficient sample sizes (> 4). Haplotype networks were constructed for each gene region and plotted on a map to infer phylogeographic patterns. Lastly, Mantel tests were performed to test for isolation by distance. It was found that 15 species occur in the Karoo and that the genus radiation in the area is in the early Palaeocene. Most diversification occurred between the late Eocene and the Miocene, coinciding with significant changes in climate. Several species show signals of demographic expansions. Isolation by distance was detected, but only with a slight correlation. It is apparent that aridification has played a vital role in the diversification of the genus in the Karoo region. This is a shared biogeographic influence between the mygalomorph fauna of the Karoo and arid region of western Australia. *Stasimopus* has radiated from the late Eocene and through the Miocene resulting in 15 extant species in the region. The Tankwa Karoo has been identified as a possible Pleistocene glacial cycle refugia for the species *S. leipoldti* . Many of the species in the Karoo are short‐range endemics, making them of high conservation concern. This study provided vital information as the Karoo is undergoing further desertification due to factors such as climate change, which may affect the future of short‐range endemic spiders.

## Graphical abstract

Aridification has played a vital role in the diversification of the *Stasimopus* genus in the Karoo region. *Stasimopus* has radiated from the late Eocene and through the Miocene resulting in 15 extant species in the region. Many of these species are short‐range endemics, leaving them at greater risk of extinction given the land‐use and climate change which will affect the Karoo in the future.

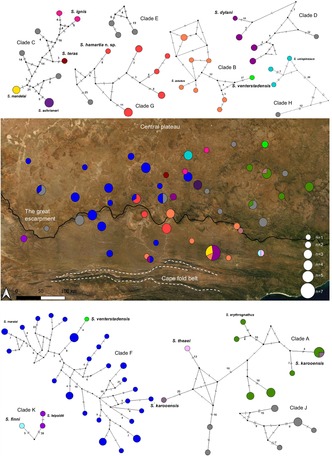

## 1 Introduction

The Karoo region of South Africa has long been neglected in studies due to the assumption of low biodiversity in semi‐arid regions (Roth‐Monzón, Mendoza‐Hernández, and Flores‐Villela 2018). This began to change in 2015 when a Strategic Environmental Assessment (SEA) was undertaken to produce a report on the state of biodiversity in the region to assist governmental policy‐makers in making informed decisions about land‐use change in the area through the Karoo BioGaps Project (Holness et al. 2016). This became imperative as in previous years pressure for land‐use change increased due to economic incentives such as mining, farming, renewable energy, intensive shale gas exploration and the Square Kilometre Array (SKA) (Sethusa 2016). To date there is still however, few studies on the faunal diversity in the area (Main et al. 2019).

The Karoo region of South Africa has a geologically rich history. The Karoo basin of South Africa in combination with four other basins globally form the Gondwanan foreland basins which were formed by collisions and terrain accretion tectonics along Gondwana's southern edge (Götz and Ruckwied 2014). The Karoo basin sedimentation dates from the late Carboniferous to the middle Jurassic (323–174 MYA) (Johnson et al. 2006). During this time the sedimentation recorded a gradual change from a glacial environment to an arid desert, as South Africa changed in latitude from the polar region to the tropics (Johnson et al. 2006; Meadows and Watkeys 1999; Tankard et al. 1982b). The Karoo basin is the only basin globally to record this 200 MY change in its sedimentation (Johnson et al. 2006). It spans 700,000 km^2^, covering 60% of South Africa's land surface (Johnson et al. 2006). Only the South Western portion of this is the modern‐day Karoo region. Due to the geological history of the basin, it is now of large economic value due to expansive coal deposits (Holness et al. 2016; Johnson et al. 2006).

**FIGURE 1:**
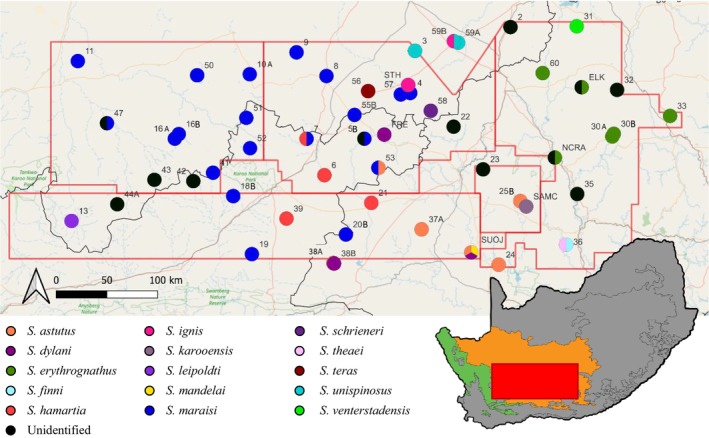
Map of the study area in the greater Karoo, in the south‐western part of South Africa. Markers indicate the 55 sites where *Stasimopus* specimens were found. If sites were near one another, they are denoted as ‘a’ and ‘b’. The localities are coloured according to the species found there. Red polygons indicate areas identified for shale gas exploration. Orange on the South African map indicates the Nama Karoo, and green indicates the succulent Karoo. Map created in qgis version 3.4.8‐Madeira (2019), available at: http://qGis.osgeo.org.

The modern‐day Karoo region of South Africa is an arid/semi‐arid area that spans one third of the land surface of South Africa (Henschel, Hoffman, and Walker 2018). The Karoo comprises two separate biomes, namely Nama Karoo and succulent Karoo, which are distinguished by climate and vegetation (Figure 1) (Henschel, Hoffman, and Walker 2018). The Nama Karoo is on the central plateau in the western portion of the country (Mucina, Rutherford, et al. 2006). It experiences unreliable summer rainfall with periods of prolonged drought (Desmet and Cowling 1999; Henschel, Hoffman, and Walker 2018). The vegetation is dominated by dwarf shrub vegetation which are xeromorphic as well as succulents in the west and grasses in the east (Desmet and Cowling 1999; Werger 1978). This is the second largest biome in South Africa covering 260,295 km^2^ (Henschel, Hoffman, and Walker 2018; Mucina, Rutherford, et al. 2006). Due to past land‐use practices approximately 60% of the biome has moderate to severe degredation of soils and vegetation (Mucina, Rutherford, et al. 2006). Only 2% of the entire Nama Karoo Biome is under conservation (Henschel, Hoffman, and Walker 2018). The succulent Karoo covers a belt from Lüderitz (Namibia) through Namaqualand and includes smaller areas of Hantam, Tanqua, Roggeveld and the little Karoo (Mucina, Jürrgens, et al. 2006). The biome experiences reliable winter rainfall (100–200 mm anually) which aids in the sustainability of vegetation (Desmet and Cowling 1999; Henschel, Hoffman, and Walker 2018). The vegetation is dominated by succulent plants and following good rains by annuals (Asteraceae and Brassicaceae) (Desmet and Cowling 1999; Werger 1978). This biome is the fourth largest in South Africa and covers 87,001 km^2^ (Henschel, Hoffman, and Walker 2018; Mucina, Jürrgens, et al. 2006). The succulent Karoo is bordered by the Cape Fold mountains, which caused isolation leading to high levels of endemisim and species richness of many taxa including hopliniid beetles, aculeate Hymenoptera, reptiles and arachnids (Milton and Dean 1999). The succulent Karoo is also the world's only entirely arid region diversity hotspot (Henschel, Hoffman, and Walker 2018; Mucina, Jürrgens, et al. 2006). Despite high levels of endemisim and being a biodiversity hotspot, only 8% of the biome is conserved (Henschel, Hoffman, and Walker 2018).

Stasimopidae is a family of trapdoor spiders endemic to southern Africa (Opatova et al. 2019). The family consists of one genus, *Stasimopus* and 56 described species (World Spider Catalog 2024). These spiders have life history traits which make it easy to detect population structuring and dispersal events increasing the likelihood of inferring phylogeographic events. Mygalomorphs are exceptionally long lived for spiders, reportedly living to 43 years old (Mason, Wardell‐Johnson, and Main 2018). Most mygalomorph spiders live in retreats comprising vertical burrows or chambers under rocks or on trees which are lined with silk (Dippenaar‐Schoeman 2002; Mason, Wardell‐Johnson, and Main 2018; Wilson et al. 2023). The females show great fidelity to their burrows, hunting from burrow entrances (Buchli 2015; Engelbrecht and Prendini 2011; Satler, Carstens, and Hedin 2013). The males are nomadic, wandering to locate females to mate with and are thus responsible for nuclear gene flow between populations. The offspring, once mature enough, will leave the mothers burrow but only move a few metres before making a new burrow to live in (Satler, Carstens, and Hedin 2013). This combination of behaviours leads to extensive population structuring and isolated geographic locations for the populations (Rix et al. 2017).

These characteristics are often associated with species which are classified as ‘short‐range endemics’, which if found to be true for the species of the genus, may make them of conservation concern. *Stasimopus* and other mygalomorph spiders are model species for phylogeographic studies due to these life history traits (Ferretti, González, and Pérez‐Miles 2014; Hedin, Starrett, and Hayashi 2012; Hedin, Carlson, and Coyle 2015; Kremen et al. 1993; Leavitt et al. 2015; Newton et al. 2020; Rix et al. 2017; Satler, Carstens, and Hedin 2013; Wilson et al. 2018). There is genetic signal present at both shallow and deeper time depths due to the low dispersal and intense population structuring typical of mygalomorph spiders (Opatova et al. 2019). Mygalomorph spiders tend to be driven to diversification due to vicariant events (Hedin, Starrett, and Hayashi 2012; Starrett et al. 2018). This makes them great candidates to link evolution over time to local geographic events. Performing this study on the *Stasimopus* genus, could possibly serve as a proxy for other mygalomorph spiders living under similar environmental conditions.

Several phylogeographic studies have been performed on Mygalomorphae species, but none have to date been performed in South Africa, nor on the *Stasimopus* genus (Beavis, Sunnucks, and Rowell 2011; Beavis and Rowell 2006; Cooper et al. 2011; Ferretti, González, and Pérez‐Miles 2014; Graham et al. 2015, 2020; Hamilton, Formanowicz, and Bond 2011; Harrison et al. 2017; Hedin, Starrett, and Hayashi 2012; Hedin, Carlson, and Coyle 2015; Hendrixson and Bond 2007; Kornilios et al. 2016; Opatova, Bond, and Arnedo 2013, 2016; Opatova et al. 2019; Rix et al. 2017; Starrett et al. 2018; Starrett and Hedin 2007; Wilson et al. 2018). Phylogeographic inference is of immense importance to conservation as there is the possibility of defining areas for conservation based on the evolutionary potential of various taxa (Rolland et al. 2011). This is particularly true for *Stasimopus* as it is expected that their evolutionary history has been shaped by vicariance and that many species in the genus are short‐range endemics. Implying that changes to the local environment has monumental implications on the genus in the Karoo. This is vital in light of the sensitivity of the Karoo ecosystem, the life history of *Stasimopus* and the drastic land‐use and climate change predicted for the area.

The aims of this study are threefold; first, to elucidate the diversification timeframe of the Karoo *Stasimopus* diversity using CO1, 16S and EF‐1ɣ genes. This information will be used to understand the diversification of the genus over geological time. Secondly, haplotype networks will be used to understand the distribution of genetic diversity in the landscape. Lastly, the phylogeographic pattern underlying the phylogeny will be evaluated and the potential drivers of the distribution of the current diversity assessed.

## 2 Materials and Methods

### 2.1 Taxon Sampling

*Stasimopus* specimens were collected from part of the Great Karoo, South Africa. The area is within the demarcated area for potential shale gas fracking. The boundary of the area is set approximately by the following coordinates: −30.88688, 26.29295 and −33.03079, 20.01661 (Figure 1). Sites were selected by the Karoo BioGaps team to cover the range of environmental conditions present in the region and within each site drainage line habitats were targeted for sampling as they are favoured by trapdoor spiders. *Stasimopus* specimens were collected at 55 sites. At each site four people spent one‐hour, thereby four man‐hours soil scraping. This methodology was effective for collecting female and juvenile specimens. In order to collect male specimens, slow driving on roads was done after rain in the evenings, which cross in search of potential mates. All specimens were preserved in a solution of cold alcohol and kept in an ice box. Specimens were later preserved in 80% ethanol in glass polytop vials for long term storage. All locality information is available in Table S1. All newly collected material was deposited in the National Collection of Arachnida (NCA) of the Agricultural Research Council (ARC), Roodeplaat, South Africa.

### 2.2 Sequence Data Generation

DNA extraction and sequencing are as stated in Brandt, Lyle, and Sole (2023) (sequencing parameters stated in Tables S2–S4). All sequences generated were assembled in CLC Bio Main Workbench Version 6.9 (https://digitalinsights.qiagen.com). All gene regions were submitted to GenBank and the accession numbers are recorded in Table S1. The CO1, 16S and EF‐1ɣ datasets were concatenated using FASconCAT v1.11 (Kück and Meusemann 2010). The edited sequences for each gene region as well as the concatenated dataset, were aligned using MAFFT online (Katoh 2005; Katoh and Toh 2008). The ‘Auto’ strategy for alignment was used in MAFFT, this inspects the direction of the sequences and adjust the alignment in correlation to the first sequence.

### 2.3 Divergence Dating

Dating the phylogeny was done using BEAST v1.8.4 (Suchard et al. 2018). All identical sequences were removed from the analysis. A fossil calibration was used to date the phylogeny. The fossil calibration points used were as follows: the tentative Hexathelidae fossil, *Rosamygale grauvogely* (Gresa‐Voltzia formation, France, Triassic) (Dunlop, Penney, and Jekel 2020; Opatova et al. 2019; Selden and Gall 1992). *Rosamygale grauvogely* is thus, the first mygalomorph appearance in the fossil record, dating to 250–240 MYA and is classified as an Avicularioidea crown‐group member (Opatova et al. 2019). The Nemesioidina clade fossil, *Cretamygale chasei* (Isle of Wight, Cretaceous) (Selden 2002), dated to 125 MYA and the fossil from the family Bemmeridae (formerly Cyrtaucheniidae), *Bolostromus destructus* (Dominican amber, Neogene) (Dunlop, Penney, and Jekel 2020; Wunderlich 1998) are assigned as the split between Bemmeridae/Theraphosidae and ‘Nemesioidina’ clades (Garrison et al. 2016; Opatova et al. 2019). Each family was represented by available sequence data from GenBank for 16S rDNA, cytochrome oxidase 1 (CO1) and elongation factor 1 gamma (EF‐1ɣ) (Table S5). Each calibration point was set by setting a hard minimum bound (the youngest possible age of the fossil based on the deposit in which it was found) and a soft upper bound (the oldest possible age). As the fossil calibration points available are only available for distant taxonomic groups, all results are to be interpreted with caution and only serve to provide a rough idea of the possible phylogeographic history for the genus in the Karoo.

The nucleotide substitution rate was determined using jModelTest v2.1.7 (Posada 2008). This was done for each gene region and the best model to account for varying base pair substitution rates was selected based on the Bayesian information criterion (BIC) (Posada 2008). A summary of the number of samples for each gene region, sequence length and substitution model are given in Table S6 (AppendixS1.) This was used to set the prior in BEAUTi v1.8.4 (Suchard et al. 2018). Other priors set included, the rate of molecular evolution to a relaxed clock with a lognormal distribution (this allows mutational rates to vary over the tree) (Michonneau 2017). As the dataset includes intraspecific and interspecific sampling the ‘Coalescent: Constant Size’ tree prior was used. An uncorrelated relaxed clock was used, as this allows for each branch of the phylogeny to have a different evolutionary rate (Drummond et al. 2006). BEAST was then run for 200,000,000 generations and sampled every 5000 generations. This was repeated twice for each analysis to ensure convergence. BEAST was run in conjunction with BEAGLE (Ayres et al. 2012).

Tracer v1.7.1 was used to confirm convergence (Rambaut et al. 2018). This was tested by checking the log file for each BEAST run and ensuring the ESS values were above 200. The individual runs were then compiled to ensure a normal distribution. Log Combiner v1.8.4 (Suchard et al. 2018) was used to combine the multiple tree files into one file. The subsampling number was set to 50,000 and the burn‐in for each tree to 50,000,000. The tree files were viewed, annotated and edited in FigTree (Rambaut 2016).

### 2.4 Demography Analyses

DNA sequence polymorphism (DnaSP) v6.12.03 (Rozas et al. 2017) was used to gather baseline statistics of gene region for each species with more than four representatives. Ambiguous nucleotides were removed, and all other settings left on default. The basic statistics recorded were as follows: nucleotide diversity (*π*), gene diversity (*H*_d_), number of haplotypes (*H*), mean number of pairwise differences (*k*) and number of segregating sites (*S*). Tajima's test and Fu and Li's tests were also run (Graham et al. 2020). Tajima's *D* compares the number of segregating sites (*S*) and the mean pairwise differences between different sequences (Ramírez‐Soriano et al. 2008; Tajima 1989). Fu and Li's D* is similar but compares the total amount of nucleotide variations to the number of singleton mutations, this was done without an outgroup (Fu and Li 1993; Ramírez‐Soriano et al. 2008). Both tests are statistical tests of neutrality and are used to determine historic population demographic changes (Graham et al. 2020; Ramírez‐Soriano et al. 2008).

### 2.5 Haplotype Networks

DnaSP was also used to prepare the sequence data for input into Network v5.0.0.0 (Fluxus‐engineering.com 2019) in order to construct haplotype networks. The 16S data was exported in Rdf format with gaps/missing data not considered, whereas the coding regions (CO1 and EF‐1ɣ) were exported with gaps considered. In order to accurately estimate the relationship between closely related *Stasimopus* individuals of the same species (which have not yet undergone speciation), a haplotype network was constructed in Network. The Network software uses the method of Maximum Parsimony to select the shortest and least complex tree. The haplotype network was constructed using the median‐joining (MJ) network algorithm. This allows for multistate data (A, T, G, C or N at any nucleotide position) (Bandelt, Forster, and Rohl 1999). All settings for network calculation were left on default. The post‐processing option of Maximum Parsimony was used in order to remove all unnecessary median vectors. Pie charts of haplotypes were plotted onto a map of the Karoo region using the coordinates of each locality. This allowed for the visualisation of the different haplotypes in geographic space.

### 2.6 Mantel Test and Isolation by Distance

A mantel test was performed to determine if geographic distance and genetic distance are correlated. This determines if there is isolation by distance. A distance matrix for the sampling localities was constructed by determining the distance between each set of coordinates using the online tool, Movable Type Scripts: Calculate distance, bearing and more between latitude/longitude points (Available at: https://www.movable‐type.co.uk/scripts/latlong.html) (Veness 2019). This tool uses the haversine formula to calculate the distance between coordinate pairs (Veness 2019). A distance matrix was constructed from the pairwise genetic distance for the CO1 dataset (as this is the most complete dataset) using Mega 7 (Kumar, Stecher, and Tamura 2016).

The Mantel test was conducted for two different datasets in R (R Core Team, 2017), using the packages ‘Ade4’ (Dray et al. 2018) and ‘vegan’ (Oksanen et al. 2019) and were both run for 10,000 permutations. First was to determine if there is genetic isolation for each locality, this thus included the 48 localities for which CO1 data is available and one representative sequence per locality, in their respective distance matrix. The second test was to test if there is isolation by distance across the various species, for this, localities which were at the edges of the species distribution were chosen across the various species. The specimens used are available in Table S7.

## 3 Results

### 3.1 Divergence Dating

**FIGURE 2:**
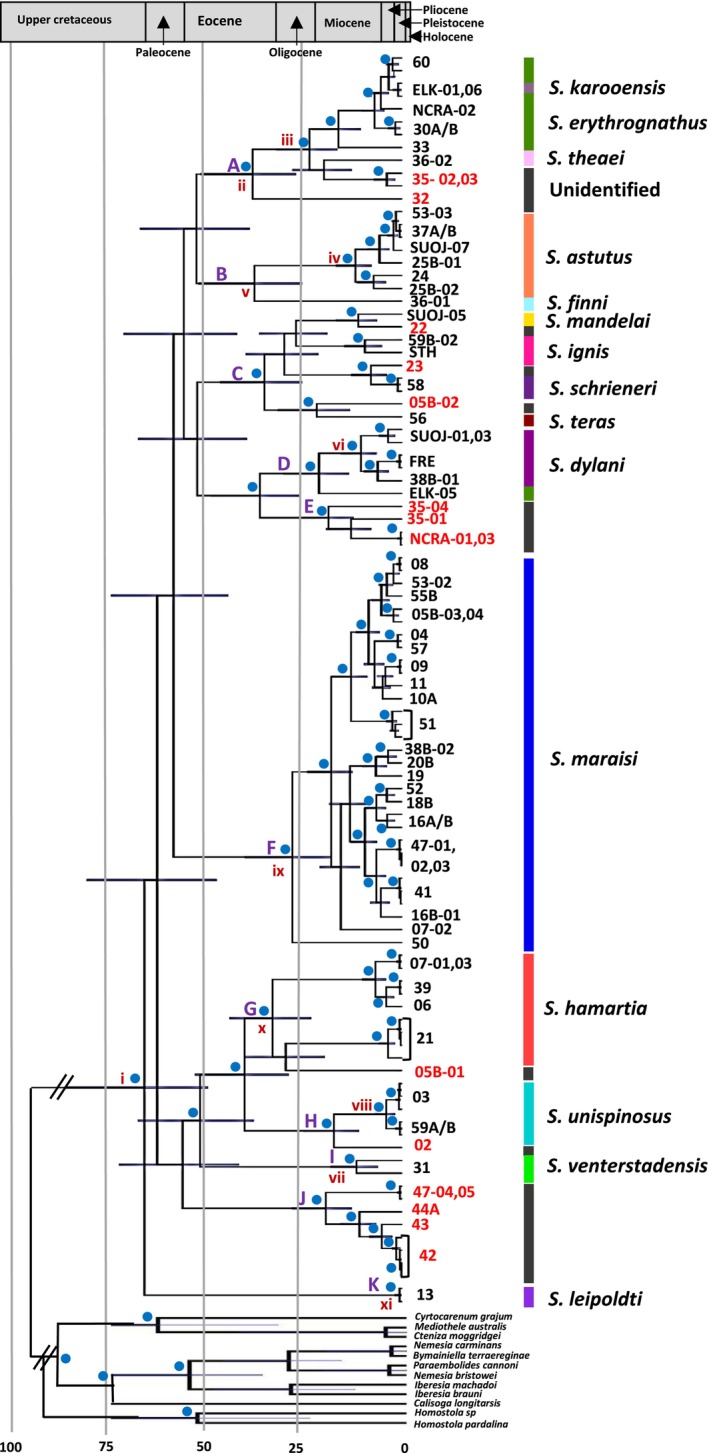
Maximum clade credibility tree from the beast analysis of CO1, 16S and EF‐1ɣ for the *Stasimopus* specimens occurring in the Karoo under the birth‐death speciation model. The grey bars indicate the confidence intervals for the dating. Outgroups available in Table S5. Posterior probabilities above 0.95 are indicated by a blue dot on the corresponding nodes. Specimen numbers correspond to localities in Figure 1, and red specimens indicate juveniles which could not be identified. Identified species are indicated by the coloured bars. Clades are numbered for the ease of discussion. Discussed nodes are indicated by roman numerals.

The dated phylogeny for the *Stasimopus* genus in the Karoo can be seen in Figure 2. Samples indicated in red text (and the grey bar on the right) could not be identified to species level as they are juveniles. Divergence estimates indicate that the genus *Stasimopus* in the Karoo dates to between 44.62–76.51 MYA, towards the beginning of the Palaeocene (Figure 2, node i).

The phylogeny is divided into 11 clades for the ease of discussion (labelled A—K). Clade A is the oldest clade. This clade is well supported and consists of a species complex of *S. erythrognathus* and *S. karooensis* as well as a single individual representing *S. theaei*. This clade is dated to 24.13–45.35 MYA (Figure 2, node ii). The age of the species complex is younger 9.36–20.12 MYA (Figure 2, node iii), the inclusion of *S. theaei* and the unidentified samples make the clade significantly older. Clade B is a well‐supported monophyletic clade, consisting of *S. astutus* and *S. finni*. The species *S. astutus* is young from the Miocene (6.89–14.74 MYA) (Figure 2, node iv), but Clade B is dated between 22.69–40.72 MYA due to the inclusion of *S. finni* (Figure 2, node v). Clade C is dated within the Oligocene, the individual species cannot be accurately dated due to small sample sizes. *Stasimopus dylani* (Clade D) (Figure 2, node vi), *S. venterstadensis* (Clade I) (Figure 2, node iiv) and *S. unispinosus* (Clade H) (Figure 2, node iiiv) are all dated to the Miocene. Clade F ( *S. maraisi* ) is one of the older species dated between 16.2–35.19 MYA (Figure 2, node ix), followed by clade G (*S. hamartia*) between 22.53–39.41 MYA (Figure 2, node x). *Stasimopus leipoldti* (Clade K) is very recently diverged in the Pleistocene (Figure 2, node xi). This may however, be inaccurate due to the small sample size and that the samples are from the same locality. Clades E and J are comprised of only unidentifiable juveniles. It is however apparent that these specimens likely comprise separate species. This dating should be interpreted with caution and any conclusion drawn is speculative due to the difficultly of dating with distantly related taxa.

### 3.2 Demographic History

**FIGURE 3:**
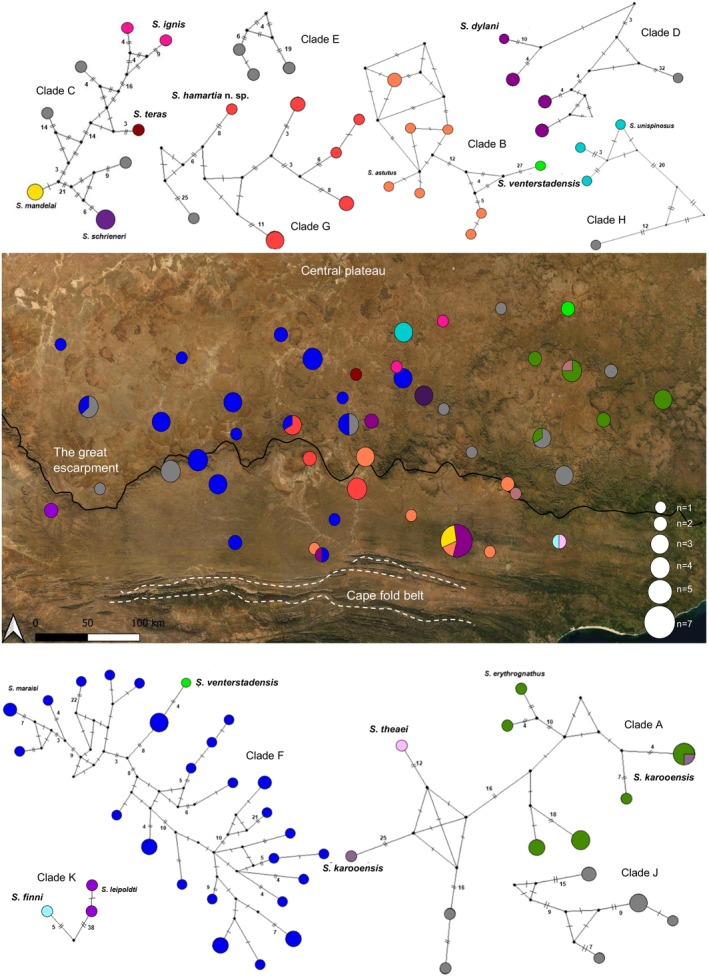
Map of the Karoo area sampled with haplotype networks for the CO1 gene region. Each circle in the networks represents a unique *Stasimopus* haplotype. The black dots on each network represents a hypothetical intermediate haplotype. Mutational steps are indicated by lines, and more than two steps by the corresponding number. The haplotype networks and map circles are proportional to the given sample size.

The haplotypes and demographic results for CO1 are presented in Figure 3 and Table 1 and the data for 16S and EF‐1ɣ in Figures S1, S2 and Tables S8, S9, respectively. The haplotype networks are largely in agreement with the phylogeny, except for the placement of the *S. venterstadensis* (Clade I, light green) individuals in the CO1 network (Figure 3) which are split and group with *S. maraisi* (Clade F, dark blue) and *S. astutus* (Clade B, coral) separately. The species complex of clade A (Figure 2) appears to be present across all three haplotype networks. The networks reveal that there is a higher degree of genetic differentiation in the CO1 gene within species than the other two gene regions, this is apparent from the higher number of mutational steps between haplotypes. EF‐1ɣ consistently has the least number of mutational steps between alleles within species. This is further substantiated by the differences in *k, S* and *π* values across the three gene regions for each species (Table 1, Tables S8 and S9). This should however be interpreted with caution as all sample sizes (except for *S. maraisi* ) are quite low.

**TABLE 1:** Demographic parameters for the CO1 gene region for the *Stasimopus* of the Karoo region. The number of sequences (*n*), nucleotide diversity (*π*), haplotypes (*H*), gene diversity (*H*_d_), mean number of pairwise differences (*k*) and segregating sites (*S*) (out of 401) are given. The results of Tajima's *D* and Fu & Li's D* are also given. The species complex referred to is that between *S. erythrognathus* and *S. karooensis.* Species with less than four representatives were omitted.

Species	*n*	*π*	*H*	*H*_d_	*k*	*S*	Tajima's *D*	Fu & Li's D*
*S. astutus*	9	0.043	7	0.917	17.222	45	0.089	1.030
*S. dylani*	7	0.048	4	0.857	19.429	41	0.472	0.794
*S. maraisi*	36	0.078	25	0.973	23.651	101	−0.682	0.419
*S. erythrognathus*	11	0.068	6	0.873	27.382	55	0.694	0.896
Species complex*	12	0.079	7	0.894	22.924	73	−0.090	0.111
*S. hamartia*	10	0.098	6	0.889	37.622	80	0.776	0.921

The haplotype diversity is high across all species considered for the CO1 gene region (Table 1). *Stasimopus unispinosus* has a lower haplotype/allele diversity for the 16S and EF‐1ɣ regions, both of 0.6 (Tables S8 and S9). Nucleotide diversity is relatively low across species and gene regions (> 0.1) (Table 1, Tables S8 and S9).

In the EF‐1ɣ network (Figure S2) the *S. maraisi* network shows a clear expansion pattern, but this is not shown in the other two gene regions. This expansion of *S. maraisi* for the EF‐1ɣ gene region is further proven by the significant negative result of the Tajima's *D* (−1.825*) and Fu & Li's D* (−3.140*) statistics. The only other significant result is for *S. hamartia* for 16S, where Fu & Li's D* is significant (1.418).

There is clear phylogeographic structuring across all three gene regions. Most species appear to be isolated to one or a few locations, except for *S. maraisi* which is widespread across the region. *Stasimopus leipoldti* was only found in one location in the Tankwa Karoo, but the type locality for the species is Clanwilliam (Western Cape), over 150 km away. A similar trend is seen for *S. erythrognathus* which was in found in the eastern Karoo, but the type locality is Worcester (Western cape), 700 km away.

There are many species occurring sympatrically at multiple locations. These include *S. maraisi* and *S. hamartia*; *S. maraisi* and *S. dylani*; *S. theaei* and *S. finni*; *S. erythrognathus* and *S. karooensis* ; *S. astutus* , *S. mandelai* and *S. dylani*; *S. maraisi* and Clade J. Clade J likely comprises a new species based on the degree of genetic variation, but no adults were found to confirm their identity.

### 3.3 Mantel Test

The Mantel tests yielded significant results for all tests conducted (*p* < 0.05). The results by locality indicate the same weak positive correlation for both software packages (Ade: *r* = 0.1413, *p* = 0.0022; Vegan: *r* = 0.1413, *p* = 0.0032). The results for the mantel test across the various species are not significant (Ade: *r* = 0.0157, *p* = 0.4333; Vegan: *r* = 0.0157, *p* = 0.437).

## 4 Discussion

This study was the first phylogeographic study of *Stasimopus* and the first of any mygalomorph spider in the Karoo region. This study has provided tentaive phylogeographic hypotheses as well as reflections on the demography of the genus.

### 4.1 Divergence Dating

From the divergence dated phylogeny, it appears that the genus *Stasimopus* radiated in the Karoo region approximately 59.63 MYA (CI: 44.62–76.51), towards the beginning of the Palaeocene. All the results discussed here should however be viewed as putative hypotheses as the divergence dating may be inaccurate. This is due to a lack of fossil representatives in more closely related taxa. The beginning of the Palaeocene is marked by the Cretaceous—Paleogene extinction event approximately 66MYA (Meadows and Watkeys 1999). The new niche gaps, along with high temperatures contributed to many radiation events, which would have aided the origin of the *Stasimopus* genus (Meadows and Watkeys 1999). The date for the radiation of *Stasimopus* in the Karoo is younger than the origin of Stasimopidae reported in Opatova et al. (2019). The family is set to have originated approximately 146 MYA, this would imply that the origin was not with any of the ancestors of the Karoo adapted species. The difference in dates could however be due to the increased taxon sampling in our study and/or the fact that the studies are dated at vastly different taxonomic levels.

Diversification of the genus appears to ramp up towards the end of the Eocene and beginning of the Oligocene (seen by the rise of clades A, B, C, G and F). This Eocene—Oligocene boundary has been noted as ‘the great divide’ due to large shifts in climate (increasing temperatures in southern Africa), leading to changes in fauna globally (Meadows and Watkeys 1999). This trend of mygalomorph diversification and geological desertification was mirrored in Australia, which saw the radiation of the Idiopidae and Halonoproctidae families at this time (Harvey et al. 2020).

The genus likely continued to radiate in the Miocene, with the rise of the rest of the clades, except K. The late Miocene coincides with an increase in temperature and onset of aridification once again (Meadows and Watkeys 1999; Tankard et al. 1982a). This is also mirrored in South Western Australia which experienced similar environmental conditions and the speciation of many plants and animals (Cooper et al. 2011; Harvey et al. 2020). The late Miocene and early Pliocene are noted as vitally important in the establishment of arid zone diversity in Australia in mygalomorph taxa such as Idiopidae (Rix et al. 2017). This same time period appears to be important for the diversification of the arid adapted *Stasimopus* species.

*Stasimopus leipoldti* (Clade K) may have arisen in the Pleistocene, this is however a tentative conclusion due to the small sample size of the species in this study as well as a lower confidence in the divergence dating. The Pleistocene saw southern Africa becoming more arid. It is also marked by alternating glacial and interglacial cycles (Barlow et al. 2013; Tankard et al. 1982a). These cycles are known to have had impacts on various species ranges (Gruber et al. 2017). There is however, evidence to suggest that Fynbos and Succulent Karoo biomes were decoupled from these effects and experienced climate stability (Potts et al. 2013). This may have led to the high levels of diversity and endemism present in these biomes today (Potts et al. 2013). *Stasimopus leipoldti* is originally described from Clanwilliam (Western Cape), over 150 km away, falling into the Succulent Karoo Biome, the samples collected for this study were from the Tankwa Karoo, which is a small, isolated enclave of this biome nestled in the harsher Nama Karoo Biome (Henschel, Hoffman, and Walker 2018). This Tankwa Karoo area may thus be a Pleistocene glacial cycle refugia for the species. A larger sample size as well as genetic sampling of the type locality is needed to show this more definitively. The same pattern of a Pleistocene glacial refugia was reported for the *Aname* genus in Pilbara, Western Australia, which experienced a similar geological history to the Karoo region (Gruber et al. 2017).

A similar relationship is seen between the type of *S. erythrognathus* (Worster, Eastern Cape) and the specimens occurring over 700 km away. This species could not be accurately dated in isolation due to forming part of a genetic species complex. The distance between the type locality and the specimens assessed here may indicate another species complex, but sampling the type locality would be required to test this.

Aridification has played a large role in shaping the evolutionary history of *Stasimopus* in the Karoo. The region is however, experiencing rapid desertification due to climate change and habitat degradation (Hoffmann 1995; Hoffmann et al. 1999). This change may thus continue to drive the diversification of the genus, or the conditions could become too harsh over too short a time span leading to the extinction of these arid adapted species. This emphasises the importance of conducting further biodiversity assessments to understand what species are truly present in the area, as well as to begin long term monitoring of the species to determine if the changing climates are causing shifts in distribution of species or in species composition as a whole.

### 4.2 Demography

The lower degree of genetic variation in the EF‐1ɣ region is expected due to this being a nuclear gene and having a lower mutational rate than the two mitochondrial genes (Allio et al. 2017). The high haplotype diversity across the various species and gene regions indicates that enough time has elapsed for diversification to occur. Majority of the species display high haplotype diversity and low nucleotide diversity across the gene regions (except for *S. unispinosus* which does not have a high haplotype diversity). This trend is indicative of a rapid expansion from a relatively small population (Beavis, Sunnucks, and Rowell 2011; Hewitt 2000). This could however also be due to the presence of crypic species. In order to gain further insight into the demographic history of these species for which only a few specimens were available, more samples are required. The incongruence of the placement of *S. venterstadensis* in the haplotype network of CO1 is potentially due to mitochondrial introgression from an ancient admixture between the two species (Croucher et al. 2004; Horoiwa et al. 2021; Kornilios et al. 2016). This could explain why the same pattern is not observed in the other gene regions, but a larger sample size of *S. venterstadensis* is required to fully explore this possibility.

The EF‐1ɣ results for *S. maraisi* indicate that the population has undergone a recent expansion event based on the negative Tajima's *D* and Fu and Li's *D* as well as the shape of the allele network (Avise 2000; Graham et al. 2015, 2020). This expansion based on the dated phylogeny likely occurred in the mid‐Miocene, coinciding with the formation of the Benguela current, leading to the Karoo experiencing a more tropical climate (Meadows and Watkeys 1999). This may have given males, which are vulnerable to desiccation, more favourable conditions to disperse further than is usually possible under arid conditions. This could have contributed to the wide species range of *S. maraisi* seen today. The influence of the Benguela current formation on accelerated speciation has previously been recorded in southern Africa. In Namibia and the Northern Cape, the formation of the Benguela current led to xeric conditions which caused local speciation events such as the formation of the aeolian sand dune system in Namibia, which in turn influenced the speciation of the *Pachysoma* dung beetles as well as *Senecio* genus of daisies (Milton et al. 2022; Sole, Scholtz, and Bastos 2005). The influence of this current on the succulent Karoo and Fynbos biomes are also well linked to speciation, such as on the tent tortoise, *Psammobates tentorius* as well as numerous plant species (Klak, Reeves, and Hedderson 2004; Verboom et al. 2009; Zhao et al. 2020). The effect of the Benguela on the Nama Karoo fauna, where *S. maraisi* is found is however largely unexplored, and requires further research.

### 4.3 Mantel Test and Geographic Influence

Individuals from localities which are in close geographic proximity tend to be more related to each other than individuals which are located further away from each other, this is supported by the results of the Mantel test. The Mantel tests showed that there is a relationship between geographic distance and genetic distance in our samples. There is a weak positive correlation in both cases, meaning that with greater geographic distance the samples are slightly less related (Diniz‐Filho et al. 2013). This is termed isolation by distance and is a common artefact in mygalomorph spiders due to their poor dispersal abilities and sedentary lifestyles (Satler, Carstens, and Hedin 2013; Thanou et al. 2017). This is important information to note given the possible fragmentation of the habitat due to land‐use changes.

This also leads to many species being considered short‐range endemics and occuring at only one or a few localities (Harvey 2002; Mason, Wardell‐Johnson, and Main 2018). This is true for many of the species considered here, except for *S. astutus* , *S. maraisi* , *S.leipoldti* and *S. erythrognathus* . Most of these species have ranges of less than 10,000 km^2^ (Harvey 2002). This is vital as this makes these species a conservation concern as they are more at risk of extinction due to climate change, habitat loss as well as habitat degredation (Gruber et al. 2017; Harvey, Johnson, and Humphreys 2011).

The species sampled appear to experience sympatry at multiple locations. This indicates that the species may have an unknown ecological niche separation or are syntopic in nature (Ríos Tamayo and Lyle 2020). Sympatry and syntopy are common in distantly related mygalomorph spiders and has been reported several times (Leavitt et al. 2015; Satler et al. 2011; Wilson et al. 2018). But in this case, the lineages are not as distantly related, posing the question of whether there is a possible niche divergence and what this may be. In the genera *Aliatypus* (North America) and *Euoplos* (Australia) the niche divergence was observed in the structure of the trapdoor lid and the habitat around the burrow (slopes and leaf litter) (Satler et al. 2011; Wilson et al. 2018). The further study of the species co‐occuring at these localities and the habitats may shed light on the speciation mechanisims within the genus.

This study provides important baseline phylogeograph hypotheses for *Stasimopus* in the Karoo. This can serve as a base for similar studies on the other mygalomorph fauna of the area or areas with similar environmental conditions.

## 5 Conclusions

*Stasimopus* is an ancient group of spiders in the Karoo region. The genus has radiated from the late Eocene and through the Miocene resulting in 15 extant species in the region. The similarities in biogeographic histories between the Karoo and arid region of western Australia have seemly affected the mygalomorph fauna in similar ways. More mygalomorph genera are needed to determine if this holds true. The Tankwa Karoo is possibly of importance as a Pleistocene glacial cycle refugia for the species *S. leipoldti* . The area should receive additional sampling to determine if it is a refugia for other taxa. Many of the species are short‐range endemics, making them of higher conservation concern. This is important to note as the Karoo is undergoing further desertification due to factors such as climate change. The genus has thrived under arid conditions, but this rapid desertification may push the various species to their adaptational limits.

## Author Contributions

**Shannon Brandt:** conceptualization (equal), data curation (lead), formal analysis (lead), funding acquisition (equal), investigation (lead), methodology (equal), project administration (lead), software (lead), validation (lead), visualization (lead), writing – original draft (lead), writing – review and editing (lead). **Robin Lyle:** data curation (supporting), funding acquisition (supporting), resources (equal), supervision (supporting), validation (supporting), writing – review and editing (supporting). **Catherine Sole:** conceptualization (equal), formal analysis (supporting), funding acquisition (equal), investigation (supporting), project administration (supporting), resources (equal), supervision (lead), validation (supporting), writing – review and editing (supporting).

## Conflicts of Interest

The authors declare no conflicts of interest.

## Supporting information


Appendix S1

Table S1

Table S5

## Data Availability

All genetic sequence data is available on NCBI Nucleotide Database (MK923763–MK935687). All specimens collected for this research have been deposited in the National Collection of Arachnida (NCA) of the Agricultural Research Council (ARC), Roodeplaat, South Africa.
