# Supplementary material for: The Phylogeography and Diversification of an Endemic Trapdoor Spider Genus, Stasimopus Simon 1892 (Araneae, Mygalomorphae, Stasimopidae) in the Karoo, South Africa
Source: Ecol Evol. 2024 Nov 24;14(11):e70621. doi: 10.1002/ece3.70621 (PMC11586105; doi:10.1002/ece3.70621)
Supplement: Supplementary file 1 — Appendix S1 [file ECE3-14-e70621-s002.docx]

**Supporting information for:**

**The phylogeography and diversification of an endemic trapdoor spider genus, *Stasimopus* Simon 1892 (Araneae, Mygalomorphae, Stasimopidae) in the Karoo, South Africa**

Table S2. Summary of primers used in this study and their corresponding gene regions.

| Gene Region | Primer name | Sequence | Primer references |
| --- | --- | --- | --- |
| CO1 | HCO2198 | 5' - TAA ACT TCA GGG TGA CCA AAA - 3' | (Folmer et al., 1994) |
|  | LCO1490 | 5' - GGT CAA CAA ATC ATA AAG ATA - 3' |  |
|  | C1-J-1751 | 5' - GGA TCA CCT GAT ATA GCA TTC CC - 3' |  |
| 16S | LRN-13398 | 5' - CGC CTG TTT AAC AAA AAC AT - 3' | (Simon et al., 1994) |
|  | LR-J-12961 | 5' - TTT AAT CCA ACA TCG AGG - 3' | (Cognato and Vogler, 2001) |
| EF-1ɣ | EF1gF78 | 5’ - ATT GCB GCN CAG TAY AGY GG - 3’ | (Ayoub et al., 2007) |
|  | EF1gR1258 | 5’ - CCT TGR TTG AAY TTC TTT CC - 3’ |  |
|  | CyrtEF1L | 5’ - CAG TTA CGC GGT ACC ACT CT - 3’ | (Kornilios et al., 2016) |
|  | CyrtEF1R | 5’ - CGC ATC TTG TCT AGC CTC TG - 3’ |  |

Table S3. The PCR protocol used for CO1 and 16S gene regions. The annealing temperature was adjusted if a sample did not amplify.

| **Phase** | **Temperature (°C)** | **Time (s)** | **40 cycles** |
| --- | --- | --- | --- |
| Denaturation | 94 | 300 |  |
|  | 94 | 15 |  |
| Annealing | 48-52 | 5 |  |
| Amplification | 72 | 15 |  |
|  | 72 | 420 |  |

Table S4. The PCR protocol used for EF-1ɣ gene region for both the initial and nested PCR. The initial PCR used the primers EF1g78 and EF1g1258 whereas the nested PCR used CyrtEF1L and CyrtEF1R. The annealing temperature was adjusted if a sample did not amplify.

|  | | | |
| --- | --- | --- | --- |
| **Phase** | **Temperature (°C)** | **Time (s)** | **35 cycles** |
| Denaturation | 94 | 180 |  |
|  | 94 | 60 |  |
| Annealing | 50 / 52 | 60 |  |
| Amplification | 72 | 60 |  |
|  | 72 | 60 |  |

Table S6. Model of nucleotide substitution selected for each gene region. The contribution of base pairs and samples by each gene region to the combined phylogeny is given. The Akaike Information Criterion (AIC) and the Bayesian Information Criterion (BIC) were both produced by jModelTest.

| **Dataset** | **Gene region** | **Base pairs** | **Number of samples** | **Percentage individuals included in phylogeny (%)** | **AIC model** | **BIC model** | **Model selected** |
| --- | --- | --- | --- | --- | --- | --- | --- |
| *Stasimopus* | CO1 | 401 | 111 | 86 | TIM1+ I+ G (I=0.379; G=0.638) | TIM1+ I+ G (I=0.379; G=0.638) | **GTR+ I+ G (I=0.379; G=0.638)** |
| *Stasimopus* | 16S | 317 | 115 | 89.15 | TIM3+ G (G=0.348) | TIM3+ G (G=0.348) | **GTR+ G**  **(G=0.348)** |
| *Stasimopus* | CO1_16S | 720 | 101 | - | TPM1uf+ I+ G (I= 0.001; G=0.299) | TPM1uf+ I+ G (I= 0.001; G=0.296) | **GTR+ G**  **(G=0.299)** |
| *Stasimopus* | EFɣ-1 | 539 | 103 | 79.84 | TPM2uf+ I+ G (I=0.519; G=0.872) | HKY+ G (G=0.231) | **HKY+ G (G=0.231)** |
| *Stasimopus* | Combined | 1257 | 129 | - | TIM1+ I+ G (I=0.531; G=0.640) | TIM1+ I+ G (I=0.531, G=0.640) | **GTR+ I+ G**  **(I=0.531, G=0.640)** |

Table S7. The locality coordinates and samples used to conduct the mantle test. The “By locality” section indicates the test that was run on 48 samples each with a unique locality to test for isolation by distance between the CO1 dataset and the geographic distances. The “By species” section indicates the subset of samples and localities tested to determine if there is a correlation between geographic distance and genetic differences across the various species.

| **By locality** | | |
| --- | --- | --- |
| **Site number** | **Specimen code** | **Species** |
| 02 | NCA 2017/1834 | Unidentified |
| 03 | NCA 2017/1835 | *S. unispinosus* |
| 04 | NCA 2017/1839 | *S. maraisi* |
| 05B | NCA 2017/1843 | Unidentified |
| 06 | NCA 2017/1849 | *S. hamartia* |
| 07 | NCA 2017/1852 | *S. hamartia* |
| 08 | NCA 2017/1855 | *S. maraisi* |
| 09 | NCA 2017/1861 | *S. maraisi* |
| 11 | NCA 2017/1866 | *S. maraisi* |
| 13 | NCA 2017/1870 | *S. leipoldti* |
| 16A | NCA 2017/1874 | *S. maraisi* |
| 16B | NCA 2017/1875 | *S. maraisi* |
| 18B | NCA 2017/1882 | *S. maraisi* |
| 19 | NCA 2017/1885 | *S. maraisi* |
| 20B | NCA 2017/1887 | *S. maraisi* |
| 21 | NCA 2017/1888 | *S. hamartia* |
| 22 | NCA 2017/1897 | Unidentified |
| 23 | NCA 2017/1899 | Unidentified |
| 24 | NCA 2017/1903 | *S. astutus* |
| 25B | NCA 2017/1906 | *S. astutus* |
| 30A | NCA 2017/1913 | *S. erythrognathus* |
| 30B | NCA 2017/1914 | *S. erythrognathus* |
| 31 | NCA 2019/610 | *S. venterstadensis* |
| 32 | NCA 2019/603 | Unidentified |
| 33 | NCA 2019/625 | *S. erythrognathus* |
| 35 | NCA 2019/620 | Unidentified |
| 36 | NCA 2019/605 | *S. finni* |
| 37A | NCA 2019/638 | *S. astutus* |
| 37B | NCA 2019/635 | *S. astutus* |
| 38B | NCA 2019/676 | *S. dylani* |
| 39 | NCA 2019/660 | *S. hamartia* |
| 41 | NCA 2019/636 | *S. maraisi* |
| 42 | NCA 2019/691 | Unidentified |
| 44A | NCA 2019/662 | Unidentified |
| 47 | NCA 2019/630 | *S. maraisi* |
| 50 | NCA 2019/689 | *S. maraisi* |
| 51 | NCA 2019/640 | *S. maraisi* |
| 52 | NCA 2019/688 | *S. maraisi* |
| 53 | NCA 2019/647 | *S. astutus* |
| 55B | NCA 2019/680 | *S. maraisi* |
| 56 | NCA 2019/643 | *S. teras* |
| 58 | NCA 2019/671 | *S. schrieneri* |
| 59B | NCA 2019/655 | *S. unispinosus* |
| 60 | NCA 2019/665 | *S. erythrognathus* |
| ELK | NCA 2019/613 | *S. erythrognathus* |
| FRE | NCA 2019/663 | *S. dylani* |
| NCRA | NCA 2017/1915 | Unidentified |
| STH | NCA 2019/642 | *S. ignis* |
| **By Species** | | |
| **Site number** | **Specimen code** | **Species** |
| 21 | NCA 2017/1888 | *S. hamartia* |
| 24 | NCA 2017/1903 | *S. astutus* |
| 60 | NCA 2019/665 | *S. erythrognathus* |
| 13 | NCA 2017/1870 | *S. leipoldti* |
| 08 | NCA 2017/1855 | *S. maraisi* |
| 19 | NCA 2017/1885 | *S. maraisi* |
| 52 | NCA 2019/688 | *S. maraisi* |
| 58 | NCA 2019/671 | *S. schrieneri* |
| 03 | NCA 2017/1835 | *S. unispinosus* |
| 06 | NCA 2017/1849 | *S. hamartia* |
| FRE | NCA 2019/663 | *S. dylani* |
| 31 | NCA 2019/610 | *S. venterstadensis* |
| 36 | NCA 2019/605 | *S. finni* |
| STH | NCA 2019/642 | *S. ignis* |
| 56 | NCA 2019/643 | *S. teras* |

Table S8. Demographic parameters for the 16S gene region for the *Stasimopus* of the Karoo region. The number of sequences (n), nucleotide diversity (π), haplotypes (H), gene diversity (H_d_), mean number of pairwise differences (k) and segregating sites (S) (out of 317) are given. The results of Tajima’s D and Fu & Li’s D* are also given. Significant results (p<0.05) are indicated by an asterisk. The species complex referred to is that between *S. erythrognathus* and *S. karooensis.* Species with less than four representatives were omitted.

| **Clade / Lineage** | **n** | **π** | **H** | **H_d_** | **k** | **S** | **Tajima’s D** | **Fu & Li's D*** |
| --- | --- | --- | --- | --- | --- | --- | --- | --- |
| *S. astutus* | 11 | 0.080 | 7 | 0.818 | 23.182 | 79 | -1.402 | -1.286 |
| *S. dylani* | 6 | 0.042 | 5 | 0.933 | 12.867 | 25 | 0.556 | 0.627 |
| *S. maraisi* | 35 | 0.068 | 23 | 0.973 | 20.225 | 70 | 0.310 | -0.108 |
| *S. erythrognathus* | 12 | 0.065 | 8 | 0.897 | 19.256 | 56 | -0.092 | 0.444 |
| Species complex* | 13 | 0.078 | 8 | 0.894 | 23.242 | 67 | -0.569 | -0.926 |
| *S. hamartia* | 11 | 0.095 | 6 | 0.800 | 28.000 | 56 | 1.850 | 1.418** |
| *S. unispinosus* | 5 | 0.006 | 2 | 0.600 | 1.800 | 3 | 1.573 | 1.572 |

Table S9. Demographic parameters for the EF-1ɣ gene region for the *Stasimopus* of the Karoo region. The number of sequences (n), nucleotide diversity (π), haplotypes (H), gene diversity (H_d_), mean number of pairwise differences (k) and segregating sites (S) (out of 539) are given. The results of Tajima’s D and Fu & Li’s D* are also given. Significant results (p<0.05) are indicated by an asterisk. The species complex referred to is that between *S. erythrognathus* and *S. karooensis.* Species with less than four representatives were omitted.

| **Clade / Lineage** | **n** | **π** | **H** | **H_d_** | **k** | **S** | **Tajima’s D** | **Fu & Li's D*** |
| --- | --- | --- | --- | --- | --- | --- | --- | --- |
| *S. astutus* | 7 | 0.014 | 7 | 1.000 | 7.330 | 21 | -0.813 | -0.651 |
| *S. maraisi* | 37 | 0.005 | 11 | 0.698 | 2.928 | 26 | -1.825** | -3.140** |
| *S. erythrognathus* | 11 | 0.006 | 7 | 0.873 | 3.309 | 10 | -0.131 | -0.478 |
| Species complex* | 12 | 0.083 | 7 | 0.833 | 3.167 | 10 | -0.180 | -0.550 |
| *S. hamartia* | 11 | 0.005 | 6 | 0.836 | 2.509 | 7 | 0.202 | -0.193 |
| *S. unispinosus* | 6 | 0.003 | 2 | 0.600 | 1.800 | 3 | 1.910 | 1.400 |

***S. venterstadensis***

***S. ignis***

***S. teras***

***S. dylani***

***S. hamartia***

***S. finni***

***S. theaei***

***S. karooensis***


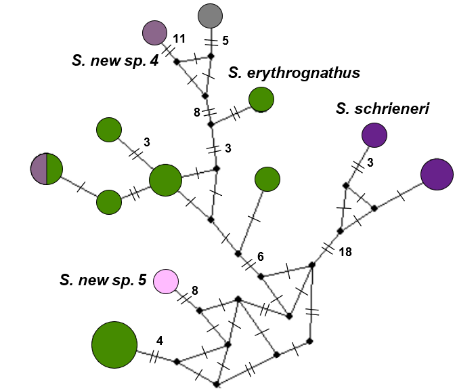

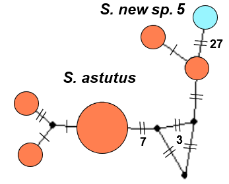

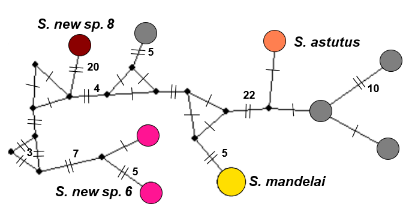

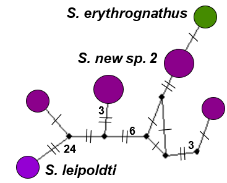

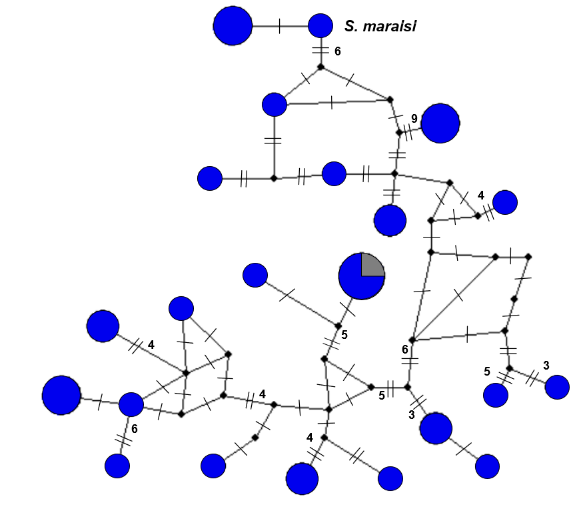

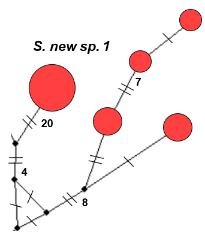

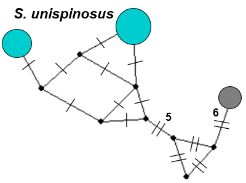

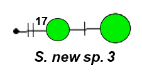

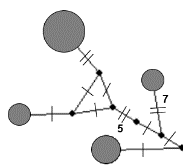


Clade C

Clade I

Clade J

Clade H

Clade G

Clade A

Clade B

Clade F

Clade D & K


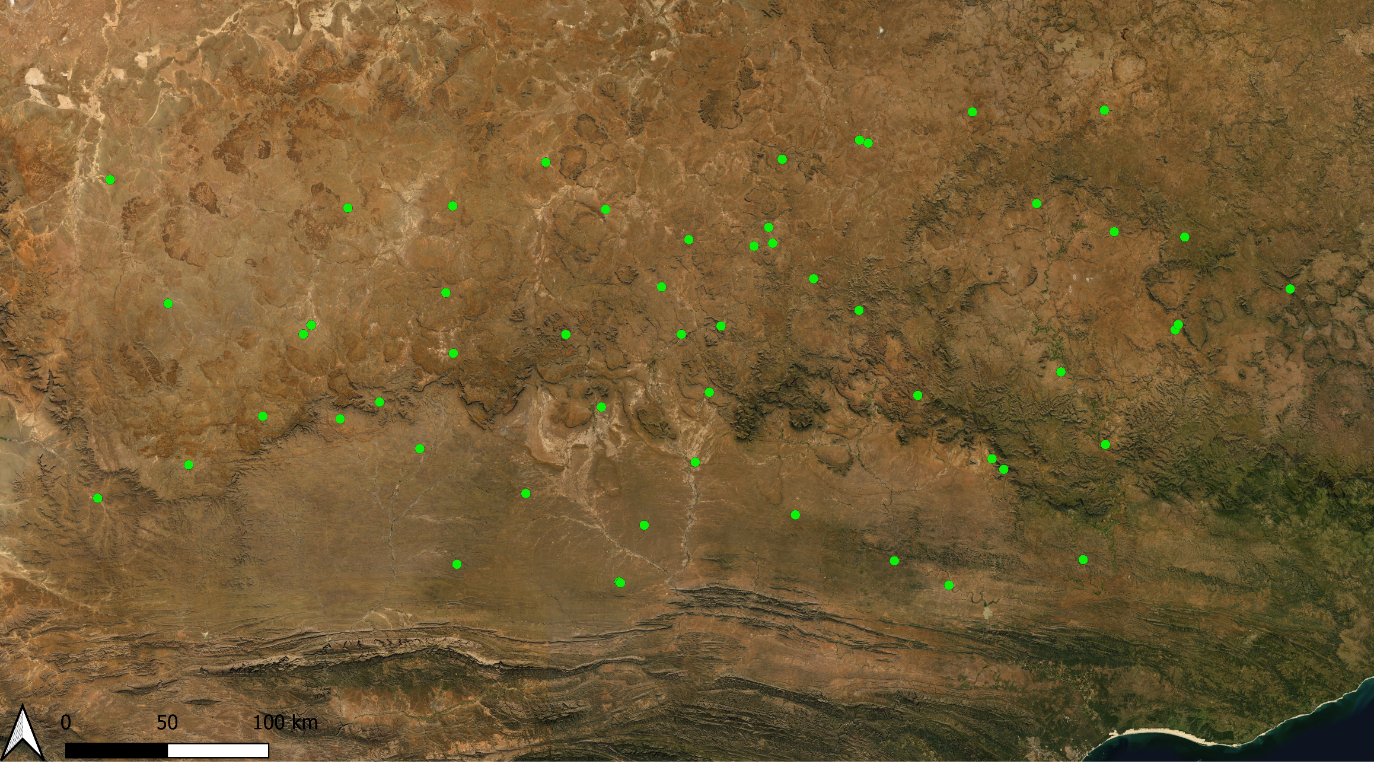


Cape fold belt

The great escarpment

Central plateau

n=1

n=2

n=3

n=4

n=5

n=6

Figure S1. Map of the Karoo area sampled with haplotype networks for the 16S gene region. Each circle in the networks represents a unique haplotype. The black dots on each network represents a hypothetical intermediate haplotype. Mutational steps are indicated by lines, and more than two steps by the corresponding number. The haplotype networks and map circles are proportional to the given sample size.

***S. venterstadensis***

***S. hamartia***

***S. dylani***

***S. ignis***

***S. teras***

***S. karooensis***


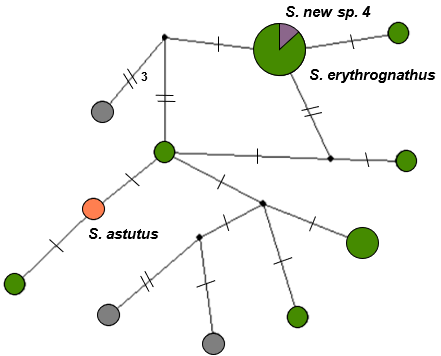

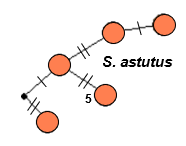

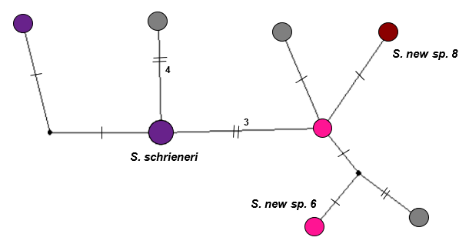

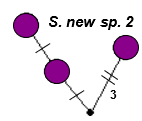

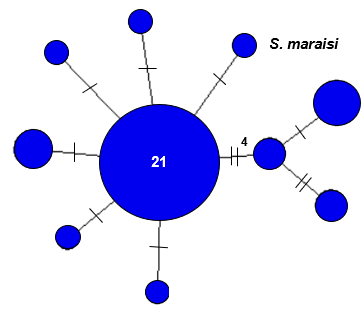

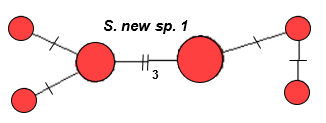

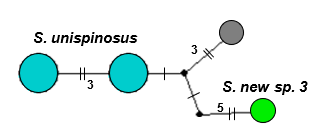

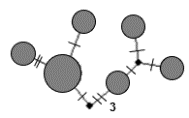

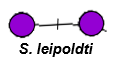


Clade C

Clade A

Clade F

Clade B

Clade K

Clade J

Clade H & I

Clade G

Clade D


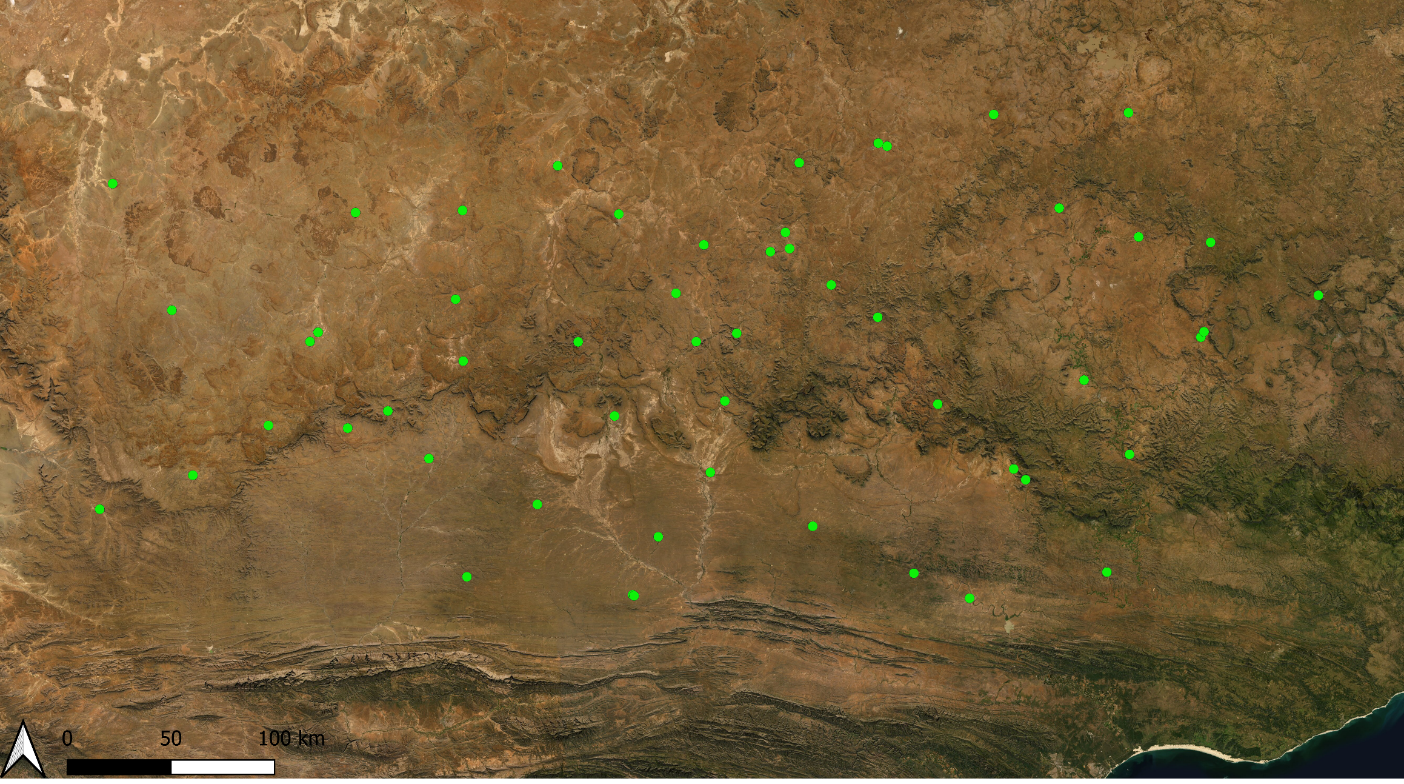


Cape fold belt

Central plateau

n=1

n=2

n=3

n=4

n=5

The great escarpment

Figure S2. Map of the Karoo area sampled with haplotype networks for the EF-1ɣ gene region. Each circle in the networks represents a unique haplotype. The black dots on each network represents a hypothetical intermediate haplotype. Mutational steps are indicated by lines, and more than two steps by the corresponding number. The haplotype networks and map circles are proportional to the given sample size.
